# Development and validation of a population-based prediction scale for osteoporotic fracture in the region of Valencia, Spain: the ESOSVAL-R study

**DOI:** 10.1186/1471-2458-10-153

**Published:** 2010-03-24

**Authors:** José Sanfélix-Genovés, Salvador Peiró, Gabriel Sanfélix-Gimeno, Vicente Giner, Vicente Gil, Manuel Pascual, Carlos Fluixá, Antonio Fuertes, Isabel Hurtado, Inmaculada Ferreros

**Affiliations:** 1 Centro Superior de Investigación en Salud Pública (CSISP), Valencia, Spain; 2 Centro de Salud de Nazaret, Departamento de Salud 5, Agencia Valenciana de Salud, Valencia, Spain; 3 Centro de Salud Ciudad Jardín, Departamento de Salud 19, Agencia Valenciana de Salud, Alicante, Spain; 4 Hospital General de Elda, Departamento de Salud 18, Agencia Valenciana de Salud, Alicante, Spain; 5 Agencia Valenciana de Salud, Valencia, Spain; 6 Centro de Salud de Valencia Benimaclet, Departamento de Salud 5, Agencia Valenciana de Salud, Valencia, Spain; 7 Centro de Salud de Alginet, Departamento de Salud 11, Agencia Valenciana de Salud, Valencia, Spain

## Abstract

**Background:**

Today, while there are effective drugs that reduce the risk of osteoporotic fracture, yet there are no broadly accepted criteria that can be used to estimate risks and decide who should receive treatment. One of the actual priorities of clinical research is to develop a set of simple and readily-available clinical data that can be used in routine clinical practice to identify patients at high risk of bone fracture, and to establish thresholds for therapeutic interventions. Such a tool would have high impact on healthcare policies. The main objective of the ESOSVAL-R is to develop a risk prediction scale of osteoporotic fracture in adult population using data from the Region of Valencia, Spain.

**Methods/Design:**

*Study design*: An observational, longitudinal, prospective cohort study, undertaken in the Region of Valencia, with an initial follow-up period of five years; *Subjects*: 14,500 men and women over the age of 50, residing in the Region and receiving healthcare from centers where the ABUCASIS electronic clinical records system is implanted; *Sources of data*: The ABUCASIS electronic clinical record system, complemented with hospital morbidity registers, hospital Accidents & Emergency records and the Regional Ministry of Health's mortality register; *Measurement of results*: Incident osteoporotic fracture (in the hip and/or major osteoporotic fracture) during the study's follow-up period. Independent variables include clinical data and complementary examinations; *Analysis*: 1) Descriptive analysis of the cohorts' baseline data; 2) Upon completion of the follow-up period, analysis of the strength of association between the risk factors and the incidence of osteoporotic fracture using Cox's proportional hazards model; 3) Development and validation of a model to predict risk of osteoporotic fracture; the validated model will serve to develop a simplified scale that can be used during routine clinical visits.

**Discussion:**

The ESOSVAL-R study will establish a prediction scale for osteoporotic fracture in Spanish adult population. This scale not only will constitute a useful prognostic tool, but also it will allow identifying intervention thresholds to support treatment decision-making in the Valencia setting, based mainly on the information registered in the electronic clinical records.

## Background

Osteoporosis is a systemic disease of the skeleton, characterized by the deterioration of the bones' macro and microstructures that leads to a loss in bone mass and the reduction in the bones' resistance increasing propensity to bone fracture [1]. At present, osteoporosis is highly prevalent, with an incidence that is rising due to the greater life expectancy of today's societies. Osteoporosis is a silent disease, yet has a major clinical impact because of its association with an increased risk of fracture. The most relevant events associated with osteoporosis are osteoporotic fractures and their consequences. Most frequently, events occur in the dorsolumbar spine, the hip and the wrist.

Hip fracture is the most serious consequence of osteoporosis. In Spain it is estimated an incidence of hip fracture in people over 50 years-old between 2-3 cases per 1,000 inhabitants/year [2-5], with a male/female distribution of 2-3:5. These figures increase considerably with age, and in subjects over the age of 60, the incidence of hip fracture is 5-7 cases per 1,000 inhabitants/year, with greater occurrence among women [6,7]. Regarding vertebral fractures, prevalence and incidence rates vary considerably, depending on the population and the criteria used to define a fracture. In subjects over the age of 50 in the Region of Asturias, Spain, prevalence was 27.2% in women and 20.8% in men [8]. In a population-based study recently conducted by our group in post-menopausal women in the city of Valencia, the prevalence of morphometric vertebral fracture was 21% in women over 50, and 46% in women over 75 [9].

According to data recently published in the Region of Valencia, Spain [10] the annual number of hospital admissions for fractures of the hip, vertebrae and forearm (fractures that are not due to major traumatism assessed using the Hospital Discharge Data Set) has increased in absolute values in the population over the age of 64. Discharges in the Region for hip fracture have gone from 3,329 in 2000 to 4,510 in 2006. Distal fractures of the forearm went from 341 to 496 during the same period. The number of vertebral fractures -that usually are managed without hospitalization- remained stable. Osteoporotic fracture has major social and health impact. Mortality for hip fracture in hospitalized patients is between 5 and 8%, and jumps to 20-30% during the first year [11]; only one third of survivors recover their pre-fracture condition.

Today, while there are effective drugs that reduce the risk of osteoporotic fracture, yet there is no broadly accepted criteria that can be used to estimate risks and decide who should receive treatment. One of the actual priorities of clinical research is to develop a set of simple and readily-available clinical data that can be used in routine clinical practice to identify patients at high risk of bone fracture, and to establish thresholds for therapeutic interventions. Such a tool would have high impact on healthcare policies. Because hip fracture is associated with the greatest disease burden, efforts are being concentrated in this area.

Studies have shown that the loss of bone mineral density (BMD), as measured by dual energy x-ray absorptiometry (DXA) is a good predictor of fracture, particularly in older women. The risk of osteoporotic fracture nearly doubles for each standard deviation decrease in BMD [12,13]. Nevertheless, there are many other factors that contribute to osteoporotic fracture, and the loss of BMD only identifies part of the risk. Studies have shown that normal BMD does not mean that there is no risk of osteoporotic fracture. Some of the Clinical Risk Factors (CRFs) involved in osteoporotic fracture only affect bone mass, and may be used to identify thresholds for testing with BMD. Other CRFs are associated with increased risk of osteoporotic fracture independently of BMD [14,15], and looking at these will improve the prediction of risk fracture [16,17]. For these reasons, different proposals have been forwarded to estimate the risk of osteoporotic fracture taking into account the different CRFs (as occurs when estimating the risk of cardiovascular events), with or without information on BMD.

This tools aims to predict the risk of an incident osteoporotic fracture during a specific period of time, usually ten years. Black et al [18], with their Fracture Index, were among the first to propose a set of criteria to predict vertebral, non-vertebral and hip fractures. More recently, Robins et al. [19], and Vazquez et al., in Spain [20], have proposed other scores although today FRAX is the scale most widely accepted internationally [21].

Robins et al. [19] developed a risk prediction scale for hip fracture at five years in post-menopausal women using data from the Women's Health Initiative study. The CRFs used in this scale include age, general health, weight, height, race, physical activity, personal history of fragility fracture after the age of 45, parental history of hip fracture, smoking habits, current use of corticoids and diabetes under treatment. Vazquez et al. [20] looked at a cohort in Rotterdam to evaluate the risk of hip and vertebral fracture at ten years in function of the subject's age and a risk score. The CRFs these authors examine are BMD (<19), history of fracture before the age of 50, parental history of hip fracture and the presence vertebral deformities. The usefulness of this index in our context has not yet been established, since it does not include treatment criteria, and still needs to be validated in the Spanish population.

The FRAX scale has been backed by the World Health Organization and its use has been rapidly widespread. It examines CRFs either independently or in combination with BMD to evaluate the risk of osteoporotic fracture at ten years, in men and women over the age of 50. The FRAX can be used to predict the probability of hip or other major osteoporotic fracture (clinical spine, hip, forearm or humerus). Predictors include age, sex, weight, height, history of prior fragility fracture, parental hip fracture, current smoking, long-term use of oral glucocorticoids, diagnosis of rheumatoid arthritis, secondary osteoporosis and alcohol consumption. The BMD of the hip is included as a non-clinical factor. The original study did not define criteria for treatment, but two later papers have used cost-utility analysis techniques to recommend treatment thresholds for the United Kingdom [22] and the United States [23]. These criteria, as the authors themselves point out, cannot be generalized to other countries with different fracture incidence rates and different healthcare costs.

The most recent score for estimating the individual risk of osteoporotic fracture or hip fracture over 10 years has been developed by Hippisley-Cox et al [24] in the UK population. These new risk prediction algorithms (QFractureScores) for osteoporotic fracture and hip fracture do not require laboratory measurement and so can be used in primary care or for individual self assessment. The validation statistics, especially for the hip fracture algorithm, suggest that the QFractureScores are likely to be useful for identifying patients at high risk of fracture in the primary care setting in the UK and showed improved performance compared with FRAX.

The main implication of these results is that when developing a scale for a specific population, or recalibrating an international scale to the fracture incidence of a given country, local data must be collected. Local studies will focus on a highly heterogeneous population that represents the real risks of a given region, and will help to establish osteoporosis treatment thresholds.

The Valencia Health Agency, the public healthcare network that covers close to 93% of the inhabitants of the Region of Valencia, has implanted an electronic medical record system called ABUCASIS. This population-based electronic database allows carrying out longitudinal studies of all the patients attending the healthcare centers from the Region of Valencia, enabling the monitoring of most relevant clinical data. It is now possible to do a low-cost cohort study that will ultimately lead to developing a risk scale with a 5-10 year window for patient follow-up.

The main objective of the ESOSVAL-R is to develop a risk prediction scale of osteoporotic fracture using data from the Region of Valencia. This scale will allow identifying intervention thresholds to support treatment decision-making in the Valencia setting, based mainly on the information registered in the electronic clinical records.

## Methods/Design

### Main objective

To develop and validate a risk prediction scale for osteoporotic fracture in the adult population of the Region of Valencia, Spain.

### Specific objectives

1. To describe baseline characteristics of the cohort under study and the prevalence of risk factors for osteoporosis. This objective will reveal the current prevalence of risk factors for osteoporosis in men and women over 50 years in the Region of Valencia.

2. To evaluate the strength of association between risk factors and the incidence of osteoporotic fracture at five years for the overall population over the age of 50, and for the five-year age-sex subgroups. This objective will also be developed specifically for the risk of hip fracture.

3. To analyze using survival models the time relationship between risk factors and the incidence of osteoporotic fracture and the risk of hip fracture.

4. To identify in patients with osteoporotic and hip fracture factors of poor prognostic for survival.

5. To develop and validate a prediction scale for the risk of osteoporotic fracture at five years, specific to the population of the Region of Valencia, Spain.

6. To evaluate the calibration and the discrimination capacity of the FRAX scale in the population of the Region of Valencia and, if necessary, to recalibrate this scale for its use in the Valencia population.

### Design

This is an observational, longitudinal, prospective cohort study with a minimum follow-up period of five years (that can be prolonged to a maximum of ten years, depending on the incidence of the most infrequent event, hip fracture). The data used in the study will be obtained primarily from the electronic clinical records (ABUCASIS system). Complementary sources of information will also be used to identify the follow-up outcomes.

### Setting

The sample will be recruited from the Region of Valencia (Spain) and, specifically, the population receiving healthcare from the Valencia Health Agency.

### Population and sample

Women and men 50 years of age and over, residing in the Region of Valencia receiving health care in centers where the ABUCASIS electronic clinical record system has been implanted. Exclusion criteria included: 1) Non-residents in the Region of Valencia, due to limitations for long-term follow-up; 2) Individuals with cognitive impairments that in the opinion of the collaborating clinicians constitutes a hindrance to collecting information about the variables used in the study; 3) People eligible for public healthcare but receiving services through private insurance companies, due to the limitations for collecting data; 4) People who are physically unable to attend their usual primary healthcare center; institutionalization is not a criterion for exclusion as long as the patient can go to the healthcare center; 5) People of Asian or African decent, because of differences in prevalence of osteoporosis due to ethnic origin. The cohort will be recruited opportunistically from among the patients who attend the collaborating primary care centers during a 15-week period.

The sample size has been calculated with the stpower Cox utility of the STATA^® ^statistics package, under the following assumptions: Alpha error (0.05), power (0.80), standard deviation for the densitometry T-score (0.75), square of the multiple correlation among co-variables (0.10), losses to follow-up of 10%, accumulated incidence of hip fracture of 15‰ after five years of follow-up and a capacity to identify as significant hazard ratios of 33% (change in the frequency of an event associated with a unit of change in the co-variable). These calculations results in a sample size of 14,122 individuals over the age of 50 in whom 180 hip fractures are expected to occur at five years of follow-up. The sample will be distributed according to the age and sex structure of the population over the age of 50 in the Region of Valencia and will be assigned in groups of 36 cases among the approximately 400 participating "practices" (two collaborating health care professionals, typically with one doctor and one nurse, per "practice") in order to be able to build a sample that is faithful to the structure of the population of the Region.

### Data sources

The main source of data will be the ABUCASIS electronic clinical records. For the outcome variables, this source will be completed with hospital morbidity records (Discharge Data sets and other systems being implanted), hospital accident and emergency records, and the regional Ministry of Health's mortality registry. Baseline data, including the patient's risk factors at the time of recruitment into the cohort, will be collected during the initial visit, using a new modified version of the ABUCASIS specifically developed to collect information about the variables used in the study that were not routinely found in clinical records. The results of previous exams (x-rays or densitometry) that may have been performed on the patients will be included in the initial evaluation (information about previous fractures and densitometry values). In the case of densitometry results, the information that will be included in the initial evaluation will be from examinations done in a period of ± 2 years at the time of recruitment.

### Main endpoint

This study's main endpoint is a major osteoporotic fracture that occurs during the follow-up period of the study. Major osteoporotic fracture is defined as a new fracture of the hip (not present at the time of recruitment), a clinical fracture of the vertebrae or a proximal fracture of the humerus or wrist. Fractures derived from severe traumatism (traffic accidents, collisions, gravitational trauma) will not be included, nor will pathological fractures due to other causes (bone cancer, metastasis to the bone, and Paget's disease established through diagnosis).

### Secondary endpoints

The study also contemplates two secondary endpoints: osteoporotic fracture of the hip and mortality due to any cause.

### Other variables

The independent variables used in this study were selected after reviewing the literature and current practice guidelines, and includes the patient's age, gender, weight and height with a calculation of the BMI, smoking habits, drinking habits, exercise habits, history of parental hip fracture, low calcium intake, untreated hypogonadism in men or women, rheumatoid arthritis, other metabolic bone diseases excluding hypogonadism, use of oral glucocorticoids, use of drugs affecting bone metabolism other than glucocorticoids, previous osteoporotic fracture, high risk of fall, prolonged immobilization, densitometric osteoporosis of the lumbar spine established through DXA (T score for L2-L4), densitometric osteoporosis of the hip established through DXA in the total hip or the femur neck (T score) and treatments for osteoporosis. The participating physicians will be trained in the management of these variables and will be given a procedures manual containing all pertinent definitions. Information about antiresorptive treatment will be collected from the GAIA system, the electronic prescription system associated with the ABUCASIS that currently covers 95% of all prescriptions dispensed in the Region. It records all the information about the drug prescribed, along with its dosage and the length of treatment.

### Development of the study

**Figure 1:**
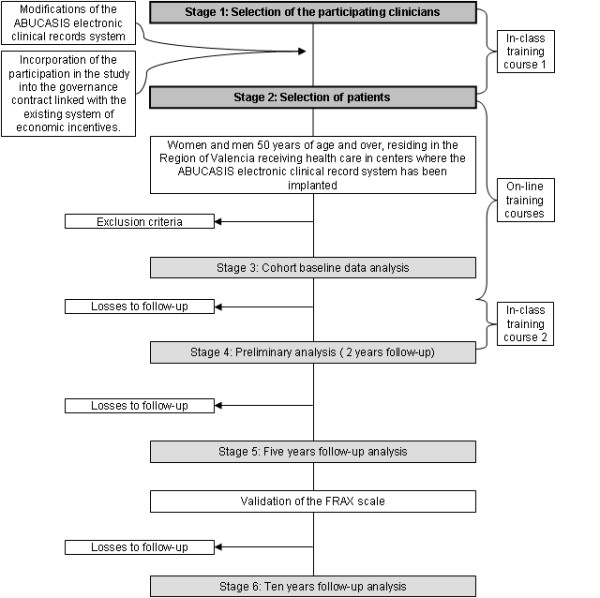
**Flow diagram of the study**.

Milestones of the study included: the selection and training of the participating clinicians, the selection of patients, the cohort baseline data analysis, the preliminary analysis and the analysis at the end of the follow up period (figure 1).

#### Selection and training of the participating clinicians

Approximately 800 clinicians will be selected for participation (family doctors and nurses) from the 23 Health Departments in the Region of Valencia which have implanted the ABUCASIS system and chose to participate voluntarily in the study. In order to stimulate involvement in ESOSVAL, the Valencia Health Agency have incorporated the participation in the study into the governance contract with the Health Departaments, and linked it to the existing system of economic incentives associated to quality indicators.

#### Selection of patients

Patients will be recruited through the participating primary healthcare centers over a 18-week period. The clinicians will select 36 patients opportunistically as they attend the medical practices, distributing them for every day of the week and according to the healthcare centers different schedules. The minimum recommended recruitment frequency is two patients per week, for the 18-week period until reaching 36 recruits with the profile (age and sex) that has been assigned to each participating clinician by the research group.

#### Follow-up

In order to develop the scales necessary to meet our research goals, follow-up must be long-term, between five to ten years, so that a high enough number of hip fractures have occurred for analysis to be significant (a fracture in any other location is much more frequent and does not require as long a follow-up period as contemplated in this study). During the initial medical visit, information will be collected and/or completed concerning all of the variables that will be used in the calculation of the risk of osteoporotic fracture. During the follow-up period, the research group will keep an exhaustive record of all new fractures, consulting all of the Valencia Health Agency's available information systems to do so. At five years (and if the researchers decide to extend the study, at ten years) the variables explored during the initial visit with the clinician will be reviewed. The fieldwork will begin in 2010, and be completed in 2015; it will be extended to 2019 if necessary.

### Ethical aspects

The study will be conducted according to the international standards of the International Guidelines for Ethical Review of Epidemiological Studies (Council for International Organizations of Medical Sciences-CIOMS-Geneva, 1991) and the recommendations of the Spanish Society of Epidemiology about the review of ethical aspects of epidemiological research.

The ESOSVAL-R study has been reviewed and approved by the Committee for Ethics and Clinical Trials of the Center for Public Health Research. The ESOSVAL-R is a naturalistic, observational study undertaken as part of routine clinical practice, with no intervention (apart from training of participating clinicians) with the patients included in the study. No additional risks associated with participation are anticipated, as no additional diagnosis, evaluation or treatment will be provided, apart from what the attending physician deems appropriate.

#### Confidentiality of the data

All information relative to the patients' identity will be considered confidential to all effects. Data concerning patients that is collected from the ABUCASIS system during the study will be documented anonymously, making impossible to use this information to identify the patients. The only connection between these data and the patient will be a simple code used exclusively for this study, in such a way that only the ABUCASIS system will be able to associate the data to an identified or identifiable individual. The data generated during the study will be handled as stipulated in the Law 5/1999 and corresponding norms. All of the researchers with access to the data used in the study will be required to sign a document guaranteeing confidentiality.

#### Informed consent

Although the study does not involve the randomization of the sample or the application of further interventions, prior to inclusion, all patients must read the "Patient Information Form" and sign a document giving consent, and granting the researchers access to information contained, in an anonymous way and only for the purposes of this study, in their clinical records.

### Statistical analysis

Once the recruiting process has been completed, the cohort baseline data will be analyzed. A description will be made of the patients' characteristics along with their current risk factors. The analysis will be stratified by five-year age-sex subgroups. The appropriate parameters (means, proportions) will be used with each variable with their corresponding 95% confidence intervals (CI95%). A bi-variable analysis will also be made to evaluate the relationships between risk factors that are of most interest. Simple Odds Ratios (OR) will be applied to estimate the strength of association and, when necessary, multiple logistic regression models will be used.

At two and five years of follow-up, an analysis will be made of the strength of association between the risk factors and the incidence of osteoporotic fracture in all locations and in the hip. For this, bi-variable analysis and Cox' proportional hazards models will be used. Additionally, a specific model will be developed to analyze the probability of death once a fracture has occurred (controlling for the effect of the different types of fractures).

Next, the most parsimonious of Cox' proportional hazards models will be applied to two-thirds of the sample (approximately 10,000 subjects randomly selected) to develop a scale to predict the risk of osteoporotic fracture. The scale's prediction capacity will be evaluated with the C statistic, and its calibration will be evaluated with the Hosmer-Lemeshow test, or a similar test. The predictive weights of the risk factors obtained in this model will be applied to the remaining third of the database (approximately 5,000 subjects) to evaluate whether prediction capacity (C statistic > 0.75) and calibration (non-significant Hosmer-Lemeshow test) are maintained. Scaling techniques will then be applied to construct a simplified scale on the basis of the validated model.

Finally, the calibration and the capacity for discrimination of the FRAX scale will be evaluated in the population of the Region of Valencia, Spain. If necessary, the FRAX will be recalibrated to make it more appropriate for the population in question. This time, the weights of the original scale will be used in conjunction with the incidence of osteoporotic fracture derived from our study. If the scale shows poor prediction capacity (statistics < 0,75), or if it is not correctly calibrated, it will be recalibrated with the weights derived from our own scale.

## Discussion

Observational studies have various inherent limitations. It is important to appraise these in an effort to minimize their effects or, at least to account for them when interpreting results. The most important limitations associated with the ESOSVAL-R study include the following:

1. Selection biases: It is virtually impossible to eliminate all biases from the selection process. First, the people most likely to visit the doctor with the greatest frequency are those who are very old or very sick and there is, thus, a greater possibility that these people will be selected for participation. Clinicians may also tend to select patients who are easier to interview (people with higher educational level), to the detriment of other candidates. In order to reduce the possibility of either of these situations' occurring, this point is specifically stressed to the clinicians during their training sessions. A selection outline, based on the healthcare centers' schedules and patient turns, has also been developed to ensure equitable selection.

2. Information biases when data is missing from the electronic clinical record: Although this problem is ever-present when a study is based on data from real clinical practices, various strategies will be applied to minimize its effect: a) It will be necessary to guarantee that all the participating clinicians are up to date on the topic of osteoporosis, that during medical visits they all respond in a similar fashion and that the quality of the electronic clinical records is as high as possible. To this end a training course will be given to all the clinicians selected for participation. These participants will be required to attend class and to complete further on-line training in the skills and knowledge necessary to manage osteoporosis. b) The electronic clinical records system has been modified to ensure that data is recorded correctly and that the registers are maintained uniformly after the recruits' initial medical visits, and during the follow-up period. These improvements in the electronic medical records system applies to all healthcare centers of the Valencia Health Agency, and not only those participating in this study.

3. Biases due to interventions and maturation: Here the risks are twofold. On the one hand, all the participating clinicians will be receiving specific training to prepare them for the study. On the other, since this is an open study, it may be affected by the doctors' learning curves throughout the process. Because of these two factors, follow-up may be more thorough in the participating patients, and they may receive better care than the population at large. This may translate into differences in the incidence of fractures in the sample cohort and in the general population which, in turn, may lead to underestimating the impact of the risk factors for fracture. Any open study is susceptible to this limitation and it's difficult to handle. However, in the case of the ESOSVAL, the training for healthcare professionals is programmed for the beginning of the study, and we hope that this will minimize the impact of this bias. Furthermore, another study will be designed in conjunction with this one to look at the patients of doctors not participating in the ESOSVAL initiative in an effort to identify possible differences in patient management.

4. Biases associated with misclassification: This bias will occur in either of two cases: when fractures of interest are not identified during the follow-up phase or, alternatively, when fractures that have not occurred during the follow-up period are improperly identified as new ones (identifying as fractures occurring during the follow-up period injuries already existing at the time of recruitment, which should have been classified as previous fractures). To limit this problem, different data sources will be used to properly identify all fractures (ABUCASIS, hospital discharge data, A&E records). Doctors will also be trained to distinguish between already existing and new fractures.

In spite of these limitations, ESOSVAL have several strengths. The study will usher in improvements in the electronic clinical records used throughout the Region and provide training to 800 healthcare professionals and this alone can have a direct impact on the quality of the clinical information registered, and on the attention given to one of today's most prevalent diseases. However, the most innovative fact of ESOSVAL is that it will use routine electronic medical records to follow-up a cohort of 14,500 subjects. Although the potential of these databases for research is obvious, no project like ESOSVAL has been conducted in Spain to date with a design like ours, or a similar number of patients and participating healthcare professionals or length of follow-up. This study will provide insight into the possibilities and limits of the use of these systems for research purposes. Finally, the ESOSVAL study will provide information about the real incidence of osteoporotic fracture in the population of the Region of Valencia (which can be useful for the rest of Spain, where there are no studies of incidence) and about the risk factors for fracture in this context. Ultimately, it will help establish a prediction scale for osteoporotic fracture. Not only will this scale constitute a useful prognostic tool, when further developed with research on cost-utility, it will help establish efficient criteria to identify the level of risk at which treatment should be initiated.

## Abbreviations

BMD: bone mineral density; DXA: dual energy x-ray absorptiometry; CRF: clinical risk factors; OR: odds ratio.

## Competing interests

The authors declare that they have no competing interests.

## Authors' contributions

JSG, SP and GSG carry out the design of the study and contributed with intellectual input in the design of this paper. JSG, VG, VG, MP, CF, AF, IH and IF contributed in several parts of the ESOSVAL Study (ABUCASIS modifications, database designs, tuition of participating clinicians). All authors contributed to the writing of the manuscript, corrected draft versions and approved the final manuscript.

## Pre-publication history

The pre-publication history for this paper can be accessed here:

http://www.biomedcentral.com/1471-2458/10/153/prepub
